# Cyclic Nanostructures of Tungsten Oxide (WO_3_)_*n*_  (*n* = 2–6) as NO_*x*_ Gas Sensor: A Theoretical Study

**DOI:** 10.1155/2014/240197

**Published:** 2014-12-02

**Authors:** Mohammad Izadyar, Azam Jamsaz

**Affiliations:** Department of Chemistry, Faculty of Sciences, Ferdowsi University of Mashhad, 9177948974 Mashhad, Iran

## Abstract

Today's WO_3_-based gas sensors have received a lot of attention, because of important role as a sensitive layer for detection of the small quantities of  NO_*x*_. In this research, a theoretical study has been done on the sensing properties of different cyclic nanoclusters of (WO_3_)_*n*_  (*n* = 2–6) for NO_*x*_  (*x* = 1,2) gases. Based on the calculated adsorption energies by B3LYP and X3LYP functionals, from the different orientations of  NO_*x*_ molecule on the tungsten oxide clusters, O–N⋯W was preferred. Different sizes of the mentioned clusters have been analyzed and W_2_O_6_ cluster was chosen as the best candidate for NO_*x*_ detection from the energy viewpoint. Using the concepts of the chemical hardness and electronic charge transfer, some correlations between the energy of adsorption and interaction energy have been established. These analyses confirmed that the adsorption energy will be boosted with charge transfer enhancement. However, the chemical hardness relationship is reversed. Finally, obtained results from the natural bond orbital and electronic density of states analysis confirmed the electronic charge transfer from the adsorbates to WO_3_ clusters and Fermi level shifting after adsorption, respectively. The last parameter confirms that the cyclic clusters of tungsten oxide can be used as NO_*x*_ gas sensors.

## 1. Introduction

Semiconducting metal oxide sensors are one of the most studied groups of the chemical sensors which have been designed to react with gases [1]. Different materials such as SnO_2_, WO_3_, ZnO, MoO_3_, TiO_2_, InO_*x*_, and mixed oxides have been studied and showed promising applications for detecting gases such as NO_*x*_, O_3_, NH_3_, CO, CO_2_, H_2_S, and SO_*x*_ [2, 3]. Therefore, improvement in the sensitivity, selectivity, rate of gas response, and reliability of oxide semiconductor gas sensors is important [4–6].

Among various metal oxide semiconductors, tungsten oxide exhibits various special properties, which makes it very promising for applications in catalysis [7, 8] and detection of the toxic gases [9]. WO_3_ based mixed oxides such as WO_3_-Ti [10], WO_3_-Pd, Pt, or Au [11], WO_3_-In_2_O_3_ [12], and WO_3_-Bi_2_O_3_ [13] have also been investigated for their sensing characteristics. These mixed oxides have been especially used in fabricating selective and sensitive NO_*x*_ gas sensors.

It was noted that different structures of WO_3_ have excellent NO_*x*_ sensing layers [9]. This is because the W ions have different oxidation state (W^6+^, W^5+^) enhancing the adsorption activity of NO_*x*_ molecule on the surface of WO_3_ structures [14, 15]. Recently, novel sensors based on tungsten oxide have been used for ozone monitoring [16, 17].

Tungsten oxide has attracted a lot of interests as an n-type oxide semiconductor [18]. WO_3_ band gap has been measured by optical absorption in the range of 2.5 to 3.2 eV and is smaller than other semiconductors [19, 20]. For WO_3_-based gas sensors, WO_3_ plays a role as a sensitive layer for detecting small quantities of NO_*x*_ and such nanoscale assemblies can achieve high sensitivity and fast response times [21].

Despite the considerable amount of works done on WO_3_ crystal structure so far, there are still important aspects of the electronic and structural properties of the WO_3_ nanoclusters and NO_*x*_ adsorption which are unclear, especially for the small clusters of WO_3_. Furthermore, the theoretical works which have been done so far are based on the standard DFT methods which seriously underestimate the semiconductor band gap. This problem can be improved by using DFT in combination with the hybrid functionals which provide a satisfactory option for the description of both the electronic and the structural properties of WO_3_ [22, 23].

In this work a theoretical procedure was applied to study the NO_*x*_  (*x* = 1,2) adsorption on the (WO_3_)_*n*_  (*n* = 2–6) nanoclusters to evaluate the reactivity of WO_3_ nanoclusters from the quantum chemistry point of view. Knowledge of the quantum reactivity indices and their role on the sensing properties of metal oxides is important to have an insight into the adsorption process and factors involved.

## 2. Computational Details

In order to study the NO_*x*_  (*x* = 1,2) adsorption on the cyclic (WO_3_)_*n*_  (*n* = 2–6) nanoclusters, theoretically, DFT method has been applied. All the calculations have been carried out by using the GAUSSIAN 09 package [24]. LANL2DZ and 6-311++G(d,p) basis sets have been applied for W and other atoms, respectively [25–27]. (WO_3_)_*n*_  (*n* = 2–6) structures were generated in the vacuum and fully optimized using two kinds of hybrid functional of B3LYP and X3LYP. The adsorption of NO and NO_2_ molecules on the clusters was investigated to evaluate some aspects of nanoclusters and NO_*x*_ interactions. Therefore, different orientations of NO_*x*_ on the nanoclusters were analyzed during these calculations. For geometry optimization, NO_*x*_ molecules were taken relaxed but the optimized structures of (WO_3_)_*n*_  (*n* = 2–6) were kept frozen. The adsorption energies of NO_*x*_ molecule and five different substrates have been computed according to

(1) $$\begin{array}{c} \Delta E=E\left[{\text{NO}}_{x}\;@\;{\left({\text{WO}}_{3}\right)}_{n}\right]\;-E{\left({\text{WO}}_{3}\right)}_{n}-E\left(\text{N}{\text{O}}_{x}\right), \end{array}$$

where *E*[NO_*x*_ @ (WO_3_)_*n*_] is the total energy of the tungsten oxide cluster-NO_*x*_ complex and *E*(WO_3_)_*n*_ and *E*(NO_*x*_) are the total energy of the isolated (WO_3_)_*n*_ and NO_*x*_, respectively.

Natural bond orbital (NBO) analysis which is suggested by Reed et al. [28, 29] was carried out to explore the distribution of the electrons into atomic and molecular orbitals. Based on these data, by HOMO-LUMO analysis, the stabilization energies were calculated. Chemical hardness, *η*, and charge transfer, Δ*N*, have been computed by Koopmans theorem [30], using the following, respectively:

(2) $$\begin{array}{c} \eta =\varepsilon_{L}-\varepsilon_{H}, \end{array}$$

(3) $$\begin{array}{c} \Delta N=\frac{\left(\mu_{B}-\mu_{A}\right)}{\left(\eta_{B}+\eta_{A}\right)}, \end{array}$$

(4) $$\begin{array}{c} \mu_{e}=\frac{\left(\varepsilon_{L}+\varepsilon_{H}\right)}{2}, \end{array}$$

where *ε*_*H*_ and *ε*_*L*_ correspond to the Kohn and Sham [31] one-electron eigenvalues associated with the frontier molecular orbitals of HOMO and LUMO, respectively. *μ*_*e*_ is the electronic chemical potential. *A* and *B* subscripts stand for NO_*x*_ and (WO_3_)_*n*_, respectively.

Densities of states (DOS) were also calculated in order to analyze the band structures and Fermi level changes of WO_3_ clusters, during the NO_*x*_ adsorption.

## 3. Results and Discussion

### 3.1. NO Adsorption on the (WO_3_)_*n*_  (*n* = 2–6) Clusters

**Figure 1:**
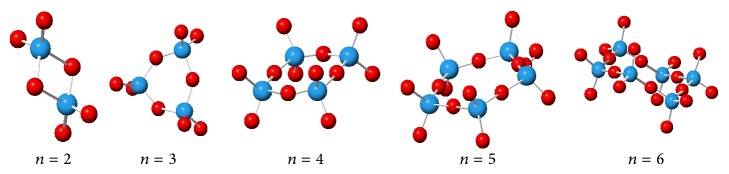
Optimized structures of the cyclic clusters of (WO_3_)_*n*_.

Figure 1 demonstrates all optimized cyclic structures of WO_3_. In order to evaluate their abilities for using as the gas sensors, cyclic WO_3_ clusters have been analyzed through the quantum chemistry approach.

**Figure 2:**
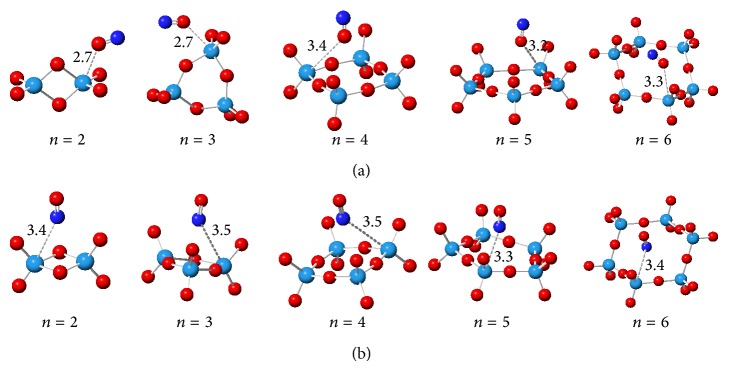
Optimized structures of NO adsorption on the (WO_3_)_*n*_ clusters at X3LYP functional: (a) through the O-head and (b) through the N-head of NO molecule.

Since there are different adsorption sites on WO_3_ clusters for NO adsorption, interfacial interactions between the clusters and NO molecule will be different from the energy point of view. The most important parameters which affect the adsorption energy are the adsorption site and NO conformation on the clusters. This means that for better analysis of the systems, it is necessary to investigate different orientations of NO molecule on the different sites of the clusters. These investigations have been illustrated in Figure 2. This figure only shows the most stable structures after optimization by X3LYP functional.

Theoretical calculations confirmed that NO adsorption through the N-head (ON⋯WO_3_) is the most stable orientations through which the most adsorption energy is obtained (Table 1).

**Figure 3:**
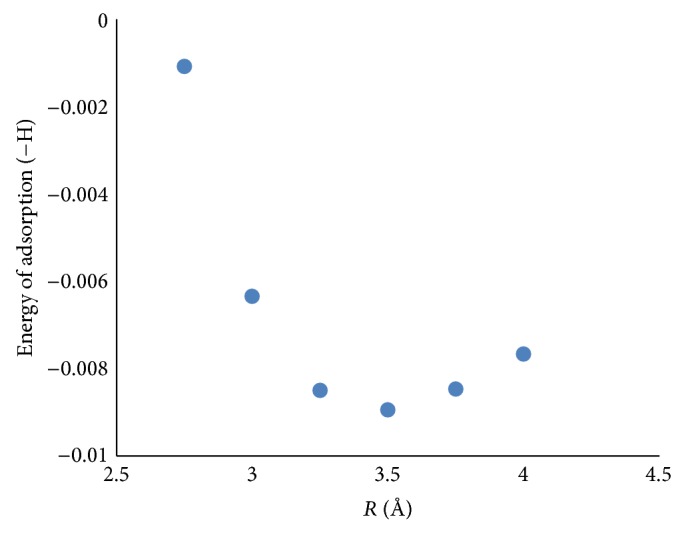
Potential energy diagram for NO-(WO_3_)_2_ system.

Figure 3 shows the potential energy diagram for NO adsorption on W_2_O_6_ clusters as an example. According to Figure 3, relaxed NO molecule is close to WO_3_ clusters from a distance of 6 Å. This figure shows the adsorption energy as a function of N⋯W distance.

Calculated adsorption energies of all studied systems have been reported in Table 1. Considering Table 1, it can be concluded that X3LYP functional predicts stronger physical adsorption than B3LYP. Correlation between the size of the cluster and adsorption energy is according to *n*: 2 > 3 ≥ 4 > 5 > 6 at the X3LYP functional. The energy of adsorption for the most stable structures of ON @ W_2_O_6_ and NO @ W_2_O_6_ complexes is 0.685 and 0.340 eV, respectively. Since ON @ W_2_O_6_ complex is a better candidate than others from the energy viewpoint, in the next stage of our study, we concentrated on this type of interaction and it has been fully investigated through the quantum chemistry approach.

Table 2 shows the quantum chemistry reactivity indices for the stable structures. According to Table 2, charge transfer and chemical hardness have been increased from NO to the surface with increase in the (WO_3_)_*n*_ clusters sizes. Higher values of the charge transfer and chemical hardness mean that increase in the size of the system makes its electronic charges unstable and lowers its flexibility.

Natural population analysis confirms that there are considerable orbital overlap between W and N atoms of the clusters and NO molecule, respectively. Natural charges for N and W atoms of NO @ W_2_O_6_ complex are +0.08, +1.5*e* before adsorption and +0.12, +1.38*e* after adsorption, respectively. This type of charge fluctuation indicates the charge flow from the adsorbate to the surface.

**Figure 4:**
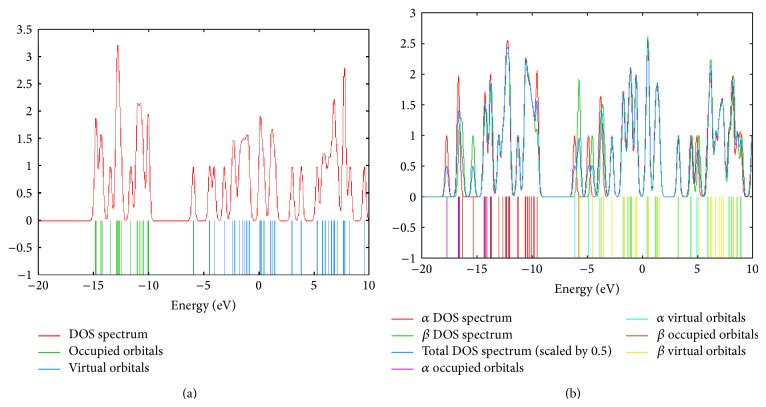
DOS spectra of W_2_O_6_ surfaces (a) before adsorption of NO and (b) after adsorption.

The influence of NO adsorption on the electronic properties of the tungsten oxide clusters was also investigated. DOS spectra of WO_3_ and ON @ WO_3_ structures have been compared. As an example, Figure 4 presents DOS spectra of W_2_O_6_ cluster before and after adsorption. Considering all systems, it was found that band gap (*E*_*g*_) changes are between 4.37 and 9.91% after the adsorption process. These results show that the adsorption of NO molecule cannot significantly affect the *E*_*g*_ and conductivity of the nanostructures while the Fermi level energy (*E*_FL_) is shifted by 0.385 eV towards the higher energies. Due to this effect, metal oxide work function (Φ) is decreased. Finally, the dispersion corrected functional, X3LYP, shows that the theoretical results which have been computed by this method differ from the B3LYP in all cases. This means that the inclusion of an empirical dispersion correction to the density functional amplifies the calculated energies of physisorption.

### 3.2. NO_2_ Adsorption on the (WO_3_)_*n*_  (*n* = 2–6) at the X3LYP Functional

**Figure 5:**
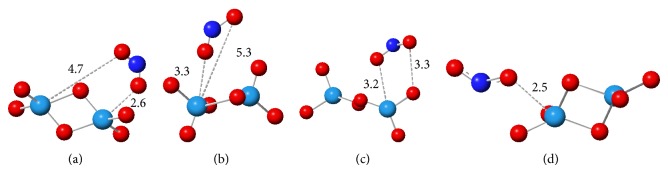
Different models of NO_2_ adsorption on W_2_O_6_.

Since, in the previous section, stronger adsorptions were obtained by X3LYP functional, this method is chosen for analysis of NO_2_ @ (WO_3_)_*n*_ systems. Four models of adsorption have been considered.  Figure 5 shows these adsorption models (a)–(d) for NO_2_ @ W_2_O_6_ complex as an example.

Adsorption energies for NO_2_ @ (WO_3_)_2_ systems have been calculated and reported in Table 3. According to Table 3, model d is the best configuration for NO_2_ adsorption from the energy point of view, 2.0 eV. This extent of adsorption energy corresponds to NO_2_ chemisorption on the W_2_O_6_ cluster. Considering all possible models, it is confirmed that the reactivity order is according to the following: d > a > c > b. Obtained correlation between the size of the cluster and adsorption energy is according to the following: *n* = 2 > 3 > 4 > 5 > 6.

Different behavior of NO_2_ adsorption on (WO_3_)_*n*_  (*n* = 2–6) has been seen in the Fermi level shifting character. As an example, W_2_O_6_ band gap is decreased from 10.10 to 9.97 eV after adsorption. Due to NO_2_ adsorption, Fermi level is shifted by 0.26 eV towards high-energy region (from −4.95 to −4.69 eV).

Charge transfer and chemical hardness changes during the NO_2_ adsorption have been calculated and reported in Table 4.

**Figure 6:**
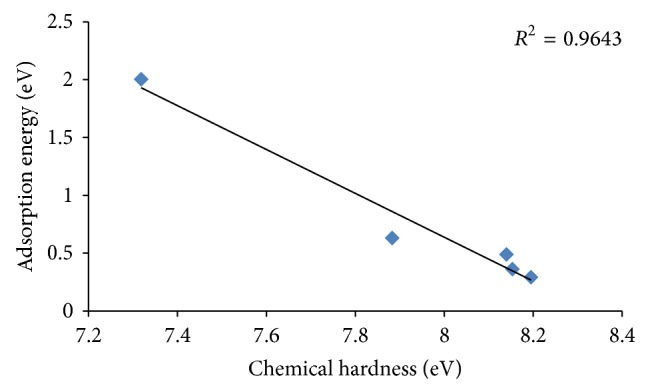
Linear correlation between the chemical hardness and adsorption energy.

**Figure 7:**
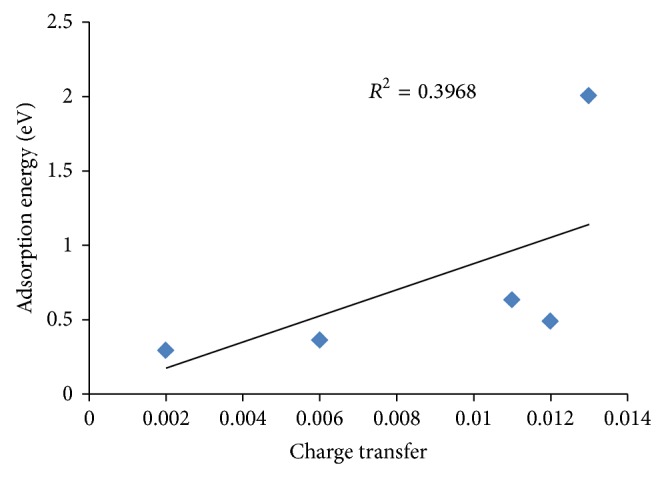
Weak linear correlation between the charge transfer parameter and adsorption energy.

According to Table 4, there is a similarity between NO_2_ and NO adsorption behavior from the chemical hardness viewpoint. Considering the electronic charge transfer, different behavior was seen. This means that increase in the size of the cluster promotes the chemical hardness while reducing the charge transfer between NO_2_ and clusters. Figures 6 and 7 show the obtained linear correlation of the adsorption energy-chemical hardness and adsorption energy-charge transfer, respectively. Good correlation between the adsorption energy and chemical hardness is obtained while this correlation is weak in the case of the charge transfer. Therefore, it can be concluded that the extent of chemical hardness fluctuation is one of the important parameters in sensing properties of the metal oxide clusters.

Natural population analysis confirms that W atom of W_2_O_6_ in the vicinity of NO_2_ molecule has a positive character and carries a charge of +1.6*e*. One O atom of NO_2_ molecule which is closer to W atom of the nanocluster has obtained more negative charge than the other. Due to this charge transfer from W, oxygen atomic charge is changed from −0.24 to −0.27*e*.

Comparing between the negative character of O atom and positive character of W atom, it can be concluded that W and O atoms do not exist as W^6+^ and O^2−^ as in the bulk of WO_3_. Therefore, we can conclude that there are considerable orbital overlaps between W and O atoms. Back-donation of lone pair electrons of O atoms to the vacant d orbitals of W atoms is the source of this covalent character for W–O bonds in the case of chemical adsorption.

## 4. Conclusions

The adsorption of NO_*x*_  (*x* = 1,2) molecules on the tungsten oxide clusters was investigated using density functional theory calculations by B3LYP and X3LYP functionals. During the adsorption of NO_*x*_ on the (WO_3_)_*n*_  (*n* = 2–6) nanoclusters, energy is released with a significant charge transfer from the NO_*x*_ to the nanoclusters. The results showed that the adsorption energy depends on the size of the cluster. X3LYP functional predicts stronger adsorption than B3LYP. W_2_O_6_ cluster is the best candidate for NO_*x*_ adsorption from the energy point of view. In order to have a meaningful understanding of molecular changes during the adsorption process, quantum chemistry reactivity indices such as chemical hardness and charge transfer parameters were evaluated. Obtained results from the natural bond orbital and electronic density of states analysis confirmed the electronic charge transfer from the adsorbates to the WO_3_ clusters and Fermi level shifting after adsorption, respectively.

## Figures and Tables

**Table 1:** Energy of adsorption for all structures (in −eV) and adsorption through O-head (N-head) of NO.

Method	W_2_O_6_	W_3_O_9_	W_4_O_12_	W_5_O_15_	W_6_O_18_
B3LYP	0.147 (0.205)	0.221 (0.244)	0.202 (0.218)	0.107 (0.194)	0.180 (0.195)
X3LYP	0.340 (0.685)	0.230 (0.276)	0.230 (0.247)	0.212 (0.220)	0.209 (0.215)

**Table 2:** Calculated quantum reactivity indices for NO adsorption.

System	*η* (eV)	Δ*N*
ON @ W_2_O_6_	7.318	0.019
ON @ W_3_O_9_	7.883	0.051
ON @ W_4_O_12_	8.190	0.059
ON @ W_5_O_15_	8.153	0.059
ON @ W_6_O_18_	8.145	0.063

**Table 3:** Calculated energy of adsorption (in −eV) for NO_2_ @ (WO_3_)_*n*_ complexes.

Model	*n* = 2	*n* = 3	*n* = 4	*n* = 5	*n* = 6
a	0.361	0.325	−0.961	0.194	0.250
b	0.192	0.172	0.158	0.124	0.109
c	0.193	0.175	0.161	0.127	0.116
d	2.003	0.631	0.489	0.362	0.292

**Table 4:** Calculated quantum chemistry reactivity indices for NO_2_ adsorption.

System	Δ*N*	*η* (eV)
NO_2_ @ W_2_O_6_	0.013	7.318
NO_2_ @ W_3_O_9_	0.011	7.883
NO_2_ @ W_4_O_12_	0.012	8.140
NO_2_ @ W_5_O_15_	0.006	8.153
NO_2_ @ W_6_O_18_	0.002	8.195
